# Pathways to food insecurity in the context of conflict: the case of the occupied Palestinian territory

**DOI:** 10.1186/s13031-022-00470-0

**Published:** 2022-07-06

**Authors:** Tracy Kuo Lin, Rawan Kafri, Weeam Hammoudeh, Suzan Mitwalli, Zeina Jamaluddine, Hala Ghattas, Rita Giacaman, Tiziana Leone

**Affiliations:** 1 Institute for Health & Aging, Department of Social and Behavioral Sciences, University of California, San Francisco, USA; 2 Middle East Centre, London School of Economics and Political Science, London, UK; 3 Institute of Community and Public Health, Birzeit University, Birzeit, The West Bank, occupied Palestinian territory; 4 Center for Research on Population and Health, American University of Beirut, Beirut, Lebanon; 5 Department of International Development, London School of Economics and Political Science, Houghton Street, London, WC2A 2AE UK

**Keywords:** Food security, Food insecurity, Food insecurity experience scale, Dietary diversity, Food consumption score, Conflict, Occupation, Occupied Palestinian territory, West Bank, Gaza Strip

## Abstract

**Background:**

Conflict reduces availability of production input and income, increases the number of days households had to rely on less preferred foods, and limits the variety of foods eaten and the portion size of meals consumed. While existing studies examine the impact of conflict on different food security measures (e.g., Food Consumption Score, Food Insecurity Experience Scale), the relationship between these measures as well as their relationship with political, economic, and agricultural factors remain under explored. Food insecurity may not only be an externality of conflict but also food deprivation may be utilized as a weapon to discourage residency in contested territories or to incentivize rebellions.

**Methodology:**

This paper examines the association between political factors (e.g., violence, policies that require permit for passage in one’s own hometown), economic factors (e.g., loss of assets, unemployment), agricultural factors (e.g., shortage of water, poor weather conditions), and food insecurity experience and dietary diversity in a conflict setting—that of the occupied Palestinian territory (oPt). The study employs generalized structural equation models to analyze the ‘Survey on socio-economic conditions for Palestinian households 2014’ dataset compiled by the Palestinian Central Bureau of Statistics—which contains a representative sample of the population in the oPt at governorate and locality levels.

**Results:**

We find that in the West Bank, residence in Area C—administered by Israel in both civil and security issues and contains illegal Israeli settlements and outposts—is associated with a higher level of agricultural hardship (*p* < 0.01) but lower economic hardship (*p* < 0.01) and a higher dietary diversity (*p* < 0.001), as compared to those living outside of Area C. In the Gaza Strip, living within one kilometer to a buffer zone is associated with lower dietary diversity (*p* < 0.01), higher level of political hardship (*p* < 0.01), and higher level food insecurity experience (*p* < 0.01) compared to not living in close proximity to a buffer zone. Concomitantly, in the Gaza Strip, food insecurity experience is associated with approximately a one-point reduction in dietary diversity as measured by the food consumption score (*p* < 0.01).

**Conclusions:**

The results suggest that broader socio-political conditions in the oPt impact different aspects of food security through augmenting the economic and agricultural hardships that are experienced by the residents. As such, it is important to address these broader political and economic structures in order to have more sustainable interventions in reducing food insecurity.

## Background

The Food and Agricultural Organization (FAO) defines food security as existing “when all people, at all times, have physical, social and economic access to sufficient, safe and nutritious food that meets their dietary needs and food preferences for an active and healthy life” [26]. The various components that are critical for ensuring food security makes it an inherently multidimensional process that involves distinct phases along a food (in)security continuum, starting with food security and ending with severe food insecurity or hunger [45, 68]. This process is complicated further in conflict-affected settings as evidence suggests that there is an endogenous relationship between conflict and severe food crisis [22, 29, 35, 44]. Not only do 60% of the world’s hungry people live in countries experiencing conflict [29] but also prevalence of undernourishment in conflict-affected low- and middle-income countries are between 1.4 and 4.4% points higher on average as compared to countries in the same income category that are not affected by conflict [38].

Directly, conflict may increase food expenditure [82], reduce diversification of the household diet [73], and decrease food security [14, [52] through conflict-associated acts, such as occupation of farmlands, destruction of livestock, and theft of crops. Indirectly, conflict impacts food insecurity through various channels, such as disrupting agricultural production [70] and affecting farmers’ investment decisions [7]. Furthermore, the relationship between conflict and food insecurity is marred by conflict-affected households often also experiencing non-conflict shocks (i.e., economic instability [46]). In these settings, households may be adopting coping strategies that may include consuming less healthful food with higher calories or less diverse diet, to survive the conflict [7, 14, 40, 70]. These coping strategies, as suggested by studies, may lead to a lower level of household dietary diversity in order to increase caloric intake and mitigate overall food insecurity.

To unpack and analyze the intricacy between conflict and aspects of food insecurity requires detailed data from conflict settings—which have been scarce. One cross-sectional study evaluates the impact of conflict on dietary diversity in Côte d’Ivoire and finds that individuals who are the direct victims of conflict and who reside in households located in the worst-hit conflict areas have lower dietary diversity [19]. Another study leverages panel survey data in Nigeria to examine the effect of the Boko Haram insurgency on food insecurity conditions and finds that insurgency reduced availability of production input and income, which then increased the number of days households had to rely on less preferred foods, limited the variety of foods eaten and the portion size of meals consumed, and reduced dietary diversity as measured by Food Consumption Score [32].

This study builds on the evidence that conflict simultaneously influences various aspects of food insecurity. While previous studies (e.g., George et al. [32]) examine the impact of conflict on different food insecurity measures simultaneously, the relationship between these different food insecurity measures have not been analyzed. Furthermore, evidence on the relationship between political, economic, and agricultural factors as well as the role each of these factors play in different pathways to food insecurity are sparse.

The aim of this study is to examine how political factors (i.e., political violence, needing a permit and submitting to checkpoints prior to passage), economic factors (i.e., loss of assets, disinvestment, restricted access to land and employment, loss of salary and income), and agricultural factors (i.e., shortage of water, bad weather conditions, damage to crops) may not only directly impact different dimensions of food insecurity but also serve as potential mediating factors in different pathways to food insecurity in a conflict-affected setting. This study poses the question: what is the relationship between political, economic, and agricultural factors and dimensions of food insecurity in a conflict-affected setting?

Understanding the impact of the interaction of political, economic, and agricutural factors on food insecurity is highly important for evidence-based, well-informed, and comprehensive policy formulation. Individuals experiencing food insecurity in conflict-affected areas require aid and humanitarian intervention. By considering only one of the aspects of food insecurity (e.g., political violence’s impact on food insecurity), policymakers may unintentionally exclude critical aspects of food insecurity and human suffering in their decision-making process. Adopting a multidimensional approach and incorporating political, economic, and agricultural factors—cognizant of their mediating role—when examining food insecurity not only may remedy the immediate problem but also may contribute to well-rounded policy prescriptions that address the root cause of the issue. This need is especially crucial in the context of a protracted conflict that has spanned decades.

The ongoing conflict in the occupied Palestinian territory (oPt)—including the West Bank and the Gaza Strip—provides a distinctive opportunity to examine the pathway to food insecurity. The prolonged occupation has led to the fragmentation of people and territory, which has been codified through military and administrative classifications (e.g., Area A, B, and C in the West Bank and buffer zones in the Gaza Strip). The occupation as well as military and administrative classifications have resulted in the formation of distinct geopolitical enclaves with variable exposures—in form and magnitude—to political and military violence. The geography of conflict in the oPt allows for analysis of differential impacts according to different levels of deprivation and conflict—a unique analysis that contributes significantly to the wider literature on conflict and food insecurity.

This study utilizes a dataset compiled from questionnaires administered at both the individual and the household level to evaluate pathways of food insecurity and how they vary according to the political and geographical division of the territory. The contribution of this study is twofold. First, the study highlights the direct impact of conflict on different aspects of food security as they interact with political, economic, and agricultural factors and evaluates the potential mediating role these factors play in the pathways to food insecurity. Second, as a sub-objective, this study adds to our understanding of the under-explored association between different dimensions of food security in conflict-affected settings and examines the relationship between two dimensions of food insecurity: food insecurity experience and dietary diversity. While this relationship has been explored in non-conflict settings, to our knowledge, there has not been a quantitative examination of such a relationship in a conflict setting.

The rest of the article is organized as follows. First, we present a theoretical framework that describes the relationship between conflict, political, economic, and agricultural factors, and aspects of food security. Next, we provide a background on the protracted conflict in the oPt. We then present descriptions on data and analytical strategy. In the last two sections, we present the results and discuss implications of the findings, which are followed by concluding remarks.

### Theoretical framework

#### Food insecurity

The FAO has specified four dimensions of food security: physical *availability* of food, physical *access* to food, food *utilization*, and *stability* of the first three dimensions over time [27]. *Availability* refers to the supply of food such as agricultural output, trade, and market distribution system. *Access* constitutes personal income and food prices. *Utilization* captures the nutritional impact of food on people and is typically measured using indicators for wasting, stunting, and low weight among children (e.g., [5, 50, 78]). *Stability* then measures the consistency of the above three dimensions and includes fluctuation in prices and supply. Indicators for *stability* can include domestic price variability. Given the objective to examine the interaction and impact of political and economic factors and how they may be associated with physical presence of food, this study focuses on the *availability* and *access* dimensions of food security.

#### Conflict and food insecurity

*Conflict* is defined and coded in numerous manners across studies and datasets [33, 82, 58, 20, 43, 47]. In order to encompass the range of conflict behavior and how different instances—however minor relatively speaking—may affect food insecurity, this study adopts the Armed Conflict Location and Event Data Set (ACLED)’s definition of conflict to represent political actions may impact food insecurity. ACLED data uses context dependent criteria to qualify an incident as an armed [2, 69].

Conflict in its different forms affects numerous aspects of demographic and socioeconomic conditions, such as policies, economy, agriculture, and health [14, 73, 82]. A critical aspect of the relationship between conflict and food insecurity is that it is an endogenous one where conflict can impact food insecurity [29], and in turn food insecurity can exacerbate conflict [22, 35]. Conflict may influence availability through disrupting agricultural production and reducing access to land (e.g., [13, 73, 82]). Conflict-related factors that may influence access include loss of businesses, farmlands, unemployment (e.g., [42]). Food insecurity and deliberating food availability have been documented as a weapon to displace individuals and deter them from returning. One example of such an occurrence is in Yemen, where conflict threatens food security for millions when humanitarian and food aid are being restricted as an instrument in conflict engagement [52]. In addition to intentional versus unintentional effects, conflict has a direct as well as an indirect association with aspects of food security. Incidences of conflict are found to reduce calories or daily energy supply [37, 77].

Focusing on the availability and access aspects of food security, this study theorizes that exposure to conflict both directly influences aspects of food security, and indirectly induces food insecurity though political, economic, and agricultural pathways—with political, economic, and agricultural factors each playing a role akin to a mediating factor not only in their respective pathway but also in all included pathways.

##### Political hardship

Those living in conflict-affected settings with greater concentration of military presence and frequent military clashes may have a higher likelihood of being exposed to disadvantageous and harmful policies and encountering barriers to a peaceful life—in addition to direct exposure to political violence (e.g., [56]) Occupying forces may implement policies such as requiring a permit and submitting individuals to checkpoints prior to passage. Such actions may lead to barriers, including inability to access markets and food sources, and increased food insecurity. For example, it is documented that during the Boko Haram attacks market operations are at times shuttered by the military [49] and transportation routes are closed [8]. These actions directly contributed to limiting the availability and access to food and increasing instances of food insecurity [80].

##### Economic hardship

Conflict may directly induce economic hardship; it may indirectly do so through generating political disadvantages and agricultural hardship, which then individually, or in combination, contribute to economic hardship [21, 70]. Living in areas that have a higher concentration of military presence directly expose individuals to economic hardships such as loss of assets [32]. Conflicts are also known to lead to disinvestment [18], hindering the economy. Additionally, conflict may indirectly influence economic hardship through policies that restrict access to work or farmland, which then lead to a loss of salary and income. Consequently, the loss of income reduces the ability to purchase food, increasing food insecurity in conflict-exposed households [14].

##### Agricultural hardship

Similarly, those living in contentious areas with greater concentration of military presence may experience a higher level of agricultural hardship. The hardship may be a direct impact of conflict such as shortage of water or damage to crops. It is documented that during the Boko Haram attacks, agricultural production was restricted in several ways, including limiting physical access to farms by Boko Haram, the regional paramilitary, or the state military [49, 74], and delaying or reducing planting or harvesting [24]. Agricultural hardship may lead to reduced income, which then lead to food insecurity. Agricultural hardship also may directly influence food insecurity when agricultural products are a household’s usual source for food consumption.

##### Relationship between political, economic, agricultural hardship, and food insecurity

In addition to their respective pathway to food insecurity, we theorize that each of these three factors play a critical role in other pathways included in this study. We reason that in conflict-affected settings these factors may influence each other and exacerbate food insecurity. For example, political hardship may impact food insecurity through generating agricultural hardship as well as economic hardship. Policies requiring a permit for basic day-to-day movement within one’s hometown may lead to limited access to land and water to farm on one’s land. Similarly, restrictions in movement that limit access to land may also limit passage to one’s employment—whether it is farming one’s land or working at a hospital in East Jerusalem. As such, the pathway between political hardship and food insecurity may be mediated by economic hardship and agricultural hardship. Likewise, the pathway between economic hardship and food insecurity may be mediated by agricultural hardship.

#### Relationship between food insecurity experience and dietary diversity in conflict settings

Studies on the relationship between conflict and food insecurity employ a range of measures and proxies for food insecurity to examine determinants of food insecurity in the context of conflict; but how these determinants may interact with each other to influence aspects of food insecurity is lesser explored. More specifically, the evidence on how food insecurity experience influence household decisions regarding food consumption and dietary diversity in the context of conflict remain sparse. The diversity of conflicts brings an additional layer of uncertainty around the potential impact of conflict on food deprivation. Level of development and intensity, length, and type of conflict all play a role in the level of food insecurity.

Conflict may influence food diversity through factors such as decline of agricultural production because of physical insecurity, lack of agricultural inputs and extension services, destruction of food processing units and food distribution system, destruction of infrastructure including roads and markets, and loss of income coupled with rising prices [19]. For example, in Côte d’Ivoire, households in the most severe conflict areas as well as individuals who are the direct victims of the conflict have lower dietary diversity [19]. Lower dietary diversity may be attributed to the lack of options in times of conflict as well as self-adjustment to food consumption patterns when exposed to conflicts. This pattern was documented in Nigeria, where households were found to respond to armed conflicts by limiting the variety of foods consumed and households make adjustments in their consumption patterns to cushion the impact of conflict-induced shocks [32]. Similarly, in Colombia, households engaged in consumption smoothing strategies, such as consuming more high caloric foods, to withstand shocks inflicted by conflicts [41].

Given the evidence that households that experience food insecurity in conflict-affected areas adopt smoothing and coping strategies, such as decreasing dietary diversity to maximize caloric intake, it is then logical to expect a negative relationship between food insecurity and dietary diversity, whereby those experiencing food insecurity have lower dietary diversity. Outside of conflict settings, dietary diversity and food insecurity experience tend to have an inverse correlation (e.g., [11, 12, 25]).

To our knowledge, there has not been a quantitative analysis that directly evaluate the relationship between food insecurity experience and dietary diversity in a conflict-affected setting. Leveraging data at the household and regional levels in the oPt, this study quantitatively examines how political and economic determinants interact to influence the relationship between food insecurity and diet diversity in a conflict setting.

##### ***Hypothesis 1***

Increased food insecurity experience is associated with reduced dietary diversity.

### Ongoing Conflict in the occupied Palestinian territory (oPt)

#### The West Bank (WB)

The ongoing conflict in the oPt poses a challenge in achieving and sustaining basic human rights. Israeli military occupation negatively affects the entire population living in the territory, internally displacing persons and disrupting the lives of 3.4 million and 1.8 million residents in the WB and Gaza Strip (GS) ([4] PCBS [62]), respectively. In the WB (5655 square kilometers), the Oslo II Accord divided the territory into three administrative divisions: Areas A, B and C. Area A is administered by the Palestinian Authority although the Israeli military periodically enters Area A to arrest and detain people. Whilst in Area B, the Palestinian Authority is responsible for ‘civil’ affairs and Israel is responsible for ‘security’. Area C is administered by Israel in both ‘civil’ and ‘security’ issues and contains illegal Israeli settlements and outposts [55]. Movement in the WB is restricted by a complex Israeli military and administrative system, which includes barriers and obstacles such as checkpoints and road obstructions. These restrictions are more pronounced in Area C, where individuals face increased restrictions in movement and in building and infrastructural development.

The protracted conflict and Israeli military occupation exert a detrimental impact on overall productivity, food production, and food availability in the WB [23, 63]. In the context of overall productivity, the military administrative system restricts the movement of the population residing in the WB and increases the time required to travel from one point to another within the WB, making otherwise normal everyday tasks time-consuming and difficult for Palestinians [79]. Concomitantly, the occupation impedes food production through restricting access to resources such as agricultural land and water. Checkpoints and continuous violence and harassment by Israeli settlers are cited obstacles to accessing agricultural land [79]. In addition, the Separation Wall also has generated agricultural hardship in the WB. The wall partitioned over 50,000 dunams of land, with only a limited number of farmers in the WB with permit to access their lands behind the wall, making it strenuous for farmers to farm their land [28]. Overall, 1.2–1.3 million dunams of land were expropriated from the Palestinian population [3]. Furthermore, Israel has full control of water resources such as aquifers in the WB—with 83% of water from these aquifers used inside Israel. Palestinians are not permitted to construct wells or water pipelines [28]. The control of and restriction on water force farmers to purchase tanked water—which raises the cost of produces and decreases profitability from farm goods [28]. The restriction of access to land and to agricultural needs lead to reduced agricultural production. The restrictions are further complicated by continuous destruction of agricultural lands, high risk of forced evictions and house destruction, and denial of permits to construct or rehabilitate homes. Relatively speaking, those living in Area C are not those without land or resources, but their resources are often constrained by Israeli policies. These policies endanger Area C communities, making the residents extremely vulnerable [54], 59]). More than 60% of the population living in Area C are characterized as food insecure [55].

The policies and resulting agricultural and economic conditions are cited as a primary factor that slow economic growth in the WB. Palestinians are unable to collect or trade their harvests because of mobility restrictions, limited external assistance and financial crisis [64]. The disruption leads to poverty and aid dependence [59]. The unemployment rate in the WB was at 14% [62] and poverty rate was at 14% in 2019 [62]. The conditions resulted in 16% of the household in WB being food insecure [55].

##### ***Hypothesis 2.1***

Residents of Area C experience higher level of food insecurity and lower level of dietary diversity, as compared to those residing outside of Area C. This hypothesis derives from the rationale that Palestinian residents of Area C may face a higher level of hardship given the more concentrated and immediate presence of the occupation forces that often lead to disruption of day-to-day life of Palestinians.

##### ***Hypothesis 2.2***

Political hardship, economic hardship and agricultural hardship are associated with higher food insecurity experience and lower dietary diversity.

#### The Gaza Strip (GS)

The prolonged siege and ongoing military operations in the GS have resulted in calamitous living conditions for the population with destructive effects on various aspects of life, including food (in)security [9]. Among key events, in December 2008, Israel launched “Operation Cast Lead” that resulted in the death of 1391 Palestinians, including 318 minors (under 18 years old). The operation caused destruction in agricultural lands and civilian homes as well as health, electricity, and water infrastructures [10]. In the GS, there are access restricted areas (ARA)—also referred to as the “buffer zones”—each of which is a 300-m-wide strip of land under Israeli control running along the border inside the Gaza Strip. These buffer zones run along the GS’s borderline and seaside; they are no-go zones for Palestinians. On the landside, buffer zones cover one kilometer into the land in GS, overlapping with agricultural areas. On the seaside, the buffer zones restrict fishing to only three nautical miles offshore.

The damaging impact of the buffer zones on food production and availability is clear. Residing in close proximity to a buffer zone puts individuals in direct physical danger—as buffer zones are continuously bombed or shot at by the Israeli military. Residing in close proximity to a buffer zone also generate agricultural hardship [1, 53]—as many of those who live near a buffer zone are farmers who own farmlands in a buffer zone. Moreover, the seaside buffer zones of three nautical miles offshore means that Palestinians can fish only within three nautical miles to the shore, effectively making 85% of the fishing zone allocated to GS off limits [28]. Fishermen crossing the buffer zones are at risk of getting shot at and arrested by the Israeli military [28]. This restriction and military decision significantly decreased not only the number of workers employed in the fisheries sector but also the amount of seafood supply for the population of the GS [61].

In addition to restrictions to land and water imposed via the buffer zones, the destruction of infrastructure and the consequent hardships also contributed to food insecurity in GS. For example, the energy shortage from infrastructure destruction has made it difficult for households to cook and store food products. The decreased capacity to store food products has led to increased expenditure associated with food and increased reliance on processed and canned food [60].

Israeli occupation, military actions, and restrictions in economic agreements have led to Palestinians food markets being dependent on Israeli food sources. As such, food prices are linked to the Israeli market—despite Palestinians’ purchasing power being six times lower than Israelis’ purchasing power [84]. This differential purchasing power results in food prices being too high for some Palestinian families to afford. As a result, about 68% of the households in the GS are food insecure [60]. Reduced capacity to farm and fish, decreased economic activity, and increased prevalence of unemployment have caused dependency on food assistance in the GS [83]. In 2014, 84% of households in the GS received assistance. Between 2013 and 2014, the number and types of assistance needed by households in the GS increased significantly; in addition to the aid and assistances that focused on cash, health insurance, and food, drinking water, clothing, and food voucher were added and received by households [31].

##### ***Hypothesis 3.1***

Residing in close proximity to a buffer zone (within one kilometer) is associated with higher level of food insecurity experience and lower level of dietary diversity.

##### ***Hypothesis 3.2***

Political hardship, economic hardship and agricultural hardship are associated with higher food insecurity experience and lower dietary diversity.

## Methods

### Data

Both conflict and food insecurity are multifaceted, thus it is important to evaluate how previously identified determinants may interact with each other to influence aspects of food insecurity in a conflict setting. Furthermore, the evidence that food insecure households in conflict-affected areas adopt specific coping strategies highlights the need to examine the relationship between food insecurity experience and dietary diversity in a conflict setting. To examine the pathways to food insecurity, this study utilizes the 2014 Socio-Economic and Food Security (SEFSec) surveys administered in the WB and the GS by the Palestinian Central Bureau of Statistics (PBCS) in coordination with the Food Security Sector (FSS). The surveys contain a representative sample of the oPt’s Palestinian population at governorate (i.e., district), locality levels (i.e., urban, rural, and Palestinian refugee camp), gender, refugee status, and for the West Bank, Areas A and B or C. Additional details to the survey data, sampling strategy, and description can be found in Socio-Economic & Food Security Survey 2014: State of Palestine [64]. The dataset for analysis combines responses from a household-level questionnaire with a sample of corresponding adults who also responded to an individual survey that includes questions on reported suffering and quality of life. The final dataset consists of 4215 WB and 2916 GS households and individual respondents—with one respondent from each included household. A total of 4193 observations from WB and 2888 observations from GS were included in the analysis; observations were only excluded due to mis-linkage between households and individuals.

#### Geopolitical setting

Israeli military occupation has led to various conflict-specific living conditions that are captured in the SEFSec dataset. The dataset contains a binary variable *Area C,* which indicates if a WB household resides in Area C or not. In the GS, there are access restricted areas (ARA)—also referred to as the “buffer zones”—which is a 300-m wide strip of land under Israeli control running along the border inside GS. The SEFSec dataset contains a binary variable *buffer zone 1000 m* that indicates if a GS household is within 1000 m—or one kilometer—to a buffer zone or not.

#### Hardships

Based on the theoretical framework on the impact of conflict on the hardships generated by conflict, we divide the documented hardships in the SEFSec dataset into three categories: political, agricultural, and economic. Each of these variables are count variables of hardships experienced. The main question measuring these hardships asks, “*In the second half of 2014, has any of the family members faced traumatic shocks*” and provides a list of 22 items**.** *Political hardship:* Different measures were used to code the political hardship variable in the WB and the GS. For the WB, three items were used (1) Israeli measure-initiated loss in assets or projects, (2) restriction imposed on access to land, and (3) lack of permits. For the GS, three additional items, unique to the GS, were added, summing up to a total of six items. The six items include three items from the WB items (listed above) and three additional items: (1) whether any member of the household was killed in the last war, (2) whether the household faced destruction or damage to their home, and (3) and whether at least one member of the family was injured in the last war. A count variable with minimum of 0 and maximum of 3 were generated for households in the WB and a count variable with minimum of 0 and maximum of 6 were generated for households in the GS. The possible numeric value for this variable counts the number of self-reported losses due to conflict captured in the survey. *Economic hardship*: Six items were summed to generate a count variable that measures economic hardships: (1) loss of assets (including land) and projects (due to non-political or unspecified reasons), (2) inability to repay loans, (3) loss of part or all of salary/ income, 4) delay of payment of salary, (5) loss of some/ all of assistance, (6) inability to pay treatment cost. These items are reasoned to be associated with direct economic resource availability. *Agricultural hardship:* A count variable was generated, summing three items: (1) shortage of water, (2) bad weather conditions (storm, inundation, drought) and (3) damage to crops (disease, failure, storage damage)**.** These items are all reasoned to be associated with agricultural production.

The Pearson pairwise correlations between these questions range from 0.002 to 0.27. Given that the survey question asked for binarized response for each item, we include a count of the total number of stressors, that is, the number of responses of “yes” from each household, for our analysis. The rationale for doing so is (1) to best capture the intensity of the stressors in households without introducing collinearity to our models by including a battery of variables relating to stress, and (2) to account for households facing multiple stressors at once.

#### Food insecurity experience

A number of instruments and scales that directly measure food insecurity based on the dimensions of food deprivation experienced by food-insecure households [16, 17, 48, 65]. In our study, we leverage the design of two instruments to generate a comparable variable. One of the experience-based scales incorporated in our study is Household Food Insecurity Access Scale (HFIAS). HFIAS is adapted from the first of the experience-based scales—the United States Household Food Security Scale Module (HFSSM)—developed in the mid-1990s and has served as the foundation for other experience-based scales. One can employ the HFIAS to assess food security levels in regions or households and monitor and evaluate the impact of programs or interventions [65]. On the other hand, the Food Insecurity Experience Scale (FIES) is designed to be used nationally, regionally, or locally and be comparable for evaluations globally [16, 17, 65, 66]. FIES measures severity of food insecurity based on each of the individuals' responses to questions about constraints on their ability to obtain adequate food. Findings can be used to identify populations or specific geographic areas that suffer from food security. FIES can be used with other indicators to identify risk factors and consequences of food insecurity—an especially relevant characteristic for our study. Since FIES directly assesses individual or household food insecurity, it can be applied in broad-based studies together with indicators of these additional aspects collected on the same units to build a better understanding of the complex phenomenon of food insecurity and to inform policy aimed at improving the well-being of the population and ending hunger [65]. The direct measure of individual's response about obtaining adequate food and its complementarity with other measures such as dietary diversity makes FIES especially advantageous for the understanding food insecurity in the population residing in the oPt.

The SEFSec survey included the HFIAS survey with nine questions regarding food insecurity. These questions are:

1. Anxiety that household will not have sufficient food (food insecurity)
2. Household members were not able to have preferred types of food due to lack of resources
3. Household members had to eat limited types of food due to lack of resources
4. Household members had to eat un-preferred food due to lack of resources
5. Household members had to eat food less than what they need because of insufficiency
6. Household members had to eat less number of meals because of insufficient food
7. Absence/ insufficient food at home because of insufficient resources to purchase
8. Any of household members had to sleep at night hungry because there was insufficient food
9. Any household member had to abstain from eating all day long because of insufficient food

Using item-response theory methods (Rasch modeling) as recommended by the FAO [57], the internal validity of the scale related to food security was assessed. We found strong correlations between items 2, 3 and 4 and between items 6 and 7 in the scale, which violates a Rasch model assumption. Consequently, item 3, which most closely resembles the wording of an item in the FIES was retained and items 2 and 4 were dropped. Similarly, item 6 was dropped from the scale due to its strong correlation with item 7.

Positive responses from the six questions were summed, and the resulting food insecurity experience scale is a 6-item scale that includes questions 1, 3, 5, 7, 8, and 9. We used this resulting six-item scale and equated it to food security data from the Palestine Gallup world poll that uses the eight-item FIES. The result shows that a score of 2 (out of 6) on the PCBS scale would equate to a score of 3 (out of 8) on the Gallup FIES scale, and would be classified as moderate food insecurity. The resulting count variable will be referred to as *food insecurity experience* and used in our models.

#### Food consumption score (FCS)

FCS has long been used by nutritionists to validate intake of necessary calories and essential nutrients [72] and has been utilized as a proxy to understand food insecurity. FCS measures dietary diversity, and its calculation is based on the World Food Program calculations in food security analysis. The food items in the questionnaire were grouped into eight food groups (i.e., main staples, pulses, vegetables, fruit, meat/fish, milk, sugar, oil) based on similarity in nutrient and caloric content. The frequency of consumption for the food items were summed into each group, the maximum frequency given was seven meaning, the value for the summed consumption frequency for food items was recorded to seven if the score was above seven. The food groups were multiplied by each weight and then summed to create the food consumption score for each household. This variable is a continuous variable.

#### Additional variables

We include variables to control for household characteristics: *head of household’s education level* and the *spouse of the head of household’s education level, household size, locality* (urban, rural, or refugee camp), and *governorate*. Given that the main outcome variable, food insecurity experience is a food deprivation measure that involves the psychological aspect of food insecurity, we reason that it is important to control for individual perception of the *sustainability* of their situation. In the SefSec survey, one question asks: *In case the situation remains as such, for how long do you think your family can sustain itself financially in the future?* The possible categories of responses are: (1) It can sustain regardless of time, (2) About one year, (3) For a few months only, (4) We barely make it, and (5) We suffer serious financial constraints, and we do not know how we can make it. We binarize the response from this question into 0, which includes response categories 2–5, indicating that the individual perceives their living situation to be unsustainable and 1, which includes the response category 1, indicating that the individual perceives their living situation to be sustainable. We also included an index for *wealth*. The wealth index calculation employs principal component analysis to calculate the weights on ownership and/or presents of household assets (e.g., type of roof, ownership or car, TV). The calculation weights the assets separately for residence (urban, rural, and refugee camps) and for the GS and the WB to account for the variation in wealth. The measure has been widely used in the past in low and middle-income settings as it shows a strong correlation with other measures such as income and expenditure [30]. *Aid* is also included in the model to control for the impact of aid in mitigating food insecurity.

### Empirical strategy

To evaluate the pathways connecting living conditions to political, economic, agricultural hardships and aspects of food insecurity we employ a generalized structural equation model. Structural equation models [51] have been used extensively in social science to examine the relationship between observed and unobserved, latent variables [36]. These models test the direct and indirect effects on theorized and pre-assumed causal relationships [66] and provide estimates on the association between these relationships. It is important to note that a structural equation model does not address causality but contributes to the understanding of the association between variables. To our knowledge, this method has not been used to evaluate the complex relationship between conflict and food insecurity. We leverage this well-suited methodology for our study and take the first step to examine the association for the theorized relationships between conflict, intermediate political, economic, and agricultural factors, and different measures of food insecurity in a conflict setting.

**Fig. 1:**
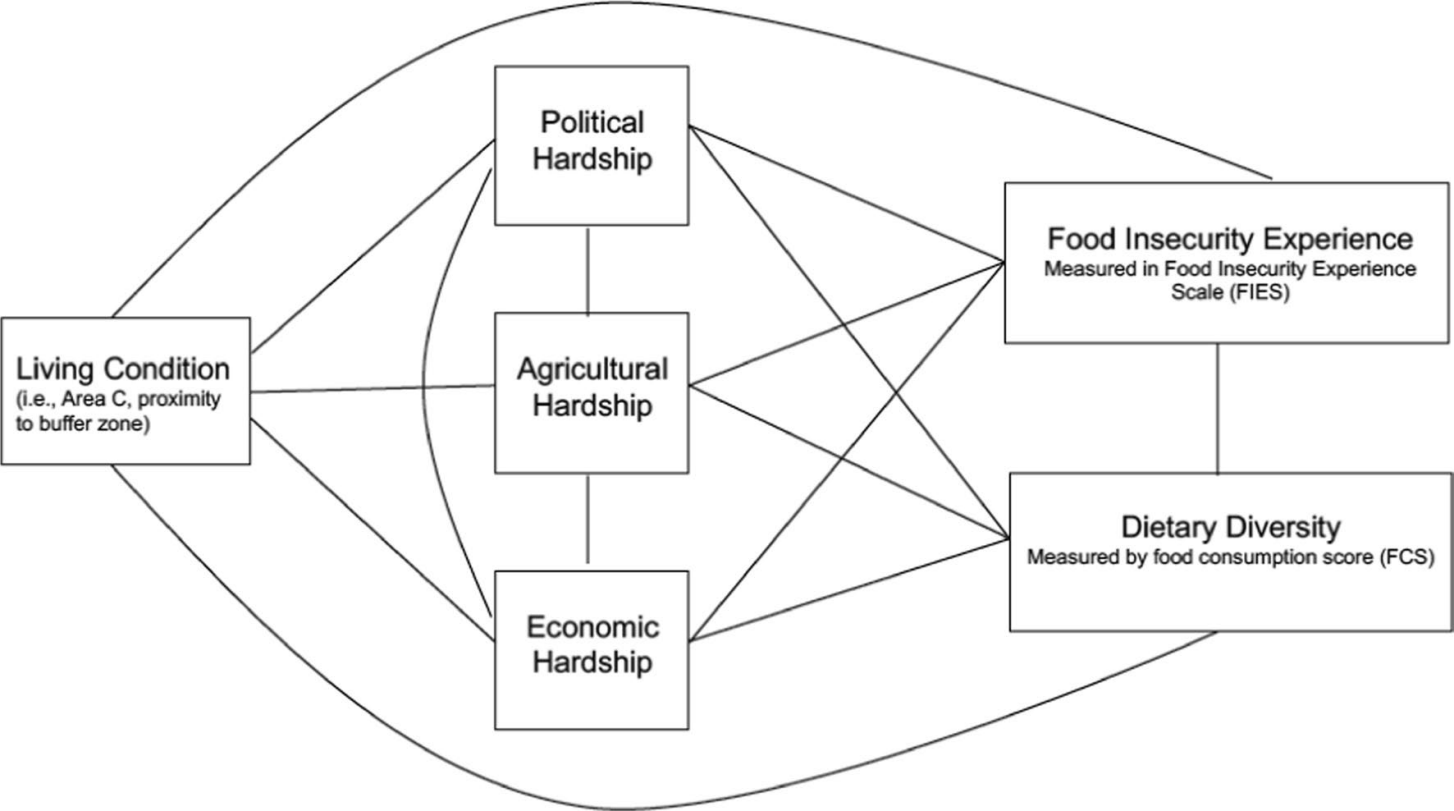
Pathway to food insecurity in the occupied palestinian territory

Structural equation models under a pathway structure were performed to analyze the SefSEC data and assess the relationship between each of the conflict settings (residency in Area C in the WB and buffer zone proximity in the GS), political, economic, and agricultural hardships, and food insecurity experience, and dietary diversity. The hypothesized pathways based on our theory are presented in Fig. 1. The analysis first examines the pathways for the association between living conditions and (1) political hardship, (2) economic hardship, and (3) agricultural hardship. Then, the analysis examines the association between political hardship, economic hardship, and agricultural hardship. Next, the pathways evaluate the association between political, economic, and agricultural hardship, and food insecurity experience and dietary diversity. Lastly the model examines, the association between food insecurity experience and dietary diversity.

Descriptive statistics were generated for all indicator variables (See Table 1). Pearson correlation analysis was conducted to examine the strength of associations between all indicator variables (See Table 2). A multilevel generalized structural equation model (GSEM) was employed to test the hypothesize model that specifies the relationship between conflict, political, economic, agricultural, geographical factors, and food insecurity and food diversity. GSEM was employed instead of a single-level structural equation model because the SefSEC dataset contains a multilevel data structure with some of the variables of interest to this study being binary variables [67, 76]. Results from GSEM provide estimates of the direct and indirect relationship of variables included in the model and estimates of error variation. STATA 14 and the function *gsem* is used to conduct the statistical calculation.

**Table 1:** Descriptive statistics for variables included in analysis

	Observations	Min	Median	Mean (SD)	Max
Food consumption score	9.004	0	79	77 (19)	112
Food insecurity experience	8.219	1	1	2 (2)	6
Agricultural hardship	8.205	0	0	0.5 (0.7)	3
Economic hardship	8.205	0	1	0.9 (1.1)	6
Political hardship	8.205	0	0	0.2 (0.4)	3
Household size	8.223	1	5	5.5 (2.6)	25
Unsustainable	9.004	0	0	0.5 (0.5)	1
Wealth index	8.216	−7.6	−0.1	−0.1 (2.1)	7.1
Aid	9.004	0	0	317 (3477)	30,000
Area C	8.211	0	1	0.9 (0.3)	1
Buffer zone proximity	8.211	0	0	0.1 (0.2)	1

**Table 2:** Pearson pairwise correlations for variables included in analysis

	Food Consumption Score	Food Insecurity Experience	Agricultural Hardship	Economic Hardship	Political Hardship	Household Size	Sustainable	Wealth Index	Aid	Area C	Buffer Zone Proximity
Food Consumption Score	1.00										
Food Insecurity Experience	−0.33	1.00									
Agricultural Hardship	−0.16	0.30	1.00								
Economic Hardship	−0.19	0.35	0.38	1.00							
Political Harship	−0.04	0.10	0.21	0.19	1.00						
Household Size	0.05	0.17	0.16	0.18	0.11	1.00					
Unsustainable	0.00	0.38	0.31	0.32	0.08	0.12	1.00				
Wealth Index	0.38	−0.39	−0.29	−0.03	−0.11	0.00	−0.36	1.00			
Aid	−0.03	0.05	0.03	0.03	0.03	0.04	0.03	−0.07	1.00		
Area C	0.00	−0.01	−0.10	0.03	−0.01	−0.03	−0.04	0.20	−0.06	1.00	
Buffer Zone Proximity	0.01	0.01	−0.04	−0.04	0.05	−0.01	−0.02	0.07	0.00	−0.16	1.00

### Limitations

The data and empirical strategy allow us to underline the relationships and interactions between conflict, political, economic, and agricultural conditions, and different aspects food security by statistically examining the association between theorized relationships, but they are limited in a couple manners. First, due to data availability at the time of analysis, we were able to only include cross-sectional survey data conducted in the oPt in 2014. The cross-sectional nature of the data prohibits the examination of causality. As such, our analyses highlight the association between our variables of interest but does not provide the direction of the causal arrows. This dataset still represents one of the richest sources of data on deprivation and food insecurity in a conflict setting. We appropriately adopt GSEMs—designed to evaluate associations between variables that have been theorized to have a relationship—to analyse the dataset. Second, as outlined, there are various dimensions for food security, and each of these dimensions are measured through different—and sometimes multiple—indicators. The analyses presented here focus on the availability and access dimensions of food security and employs the derived experienced-based food insecurity scale and FCS as indictors. We recognize the importance of other dimensions of food security on a complex continuum. However, to capture these additional dimensions is outside the scope of our study, which focuses on the physical presence of food. Furthermore, inclusion of additional dimensions will require data that are not available at the time of analysis. Our indicators of choice have their shortcomings, such as being self-reported and potentially biased measures, but they are collected using validated instruments that can be adopted globally. To our knowledge, they are the best available suited measurements for capturing the availability and access dimensions of food security. Nevertheless, we hope to incorporate additional aspects of food security in future studies.

## Results

### The West Bank

We first present the findings from the WB GSEM analysis in Table 3. Focusing on the pathway for dietary diversity (Model 1), we find that food insecurity experience is negatively associated with dietary diversity as measured by Food Consumption Score (*p* < 0.01); a unit increase in food insecurity experience is associated with approximately one point reduction (natural log of −0.035) in Food Consumption Score. The finding suggests that those with food insecurity experience are also more likely to experience lower dietary diversity and provide preliminary evidence to support our hypothesis that households experiencing food insecurity engage in coping mechanisms, such as lower the number of options in their diet. The relationships between other variables are consistent with current literature. Lower dietary diversity is associated with higher level of economic hardship (*p* < 0.01) and agricultural hardship (*p* < 0.05), female head of household (*p* < 0.05), and residence in a refugee camp (*p* < 0.01). On the other hand, more diverse diet in the WB is associated with head of household (*p* < 0.05) and spouse with post-secondary education (compared with secondary education as the reference group) (*p < 0.01*), larger household size (*p* < 0.01), increased household wealth (*p* < 0.01), and residence in Area C.

**Table 3:** GSEM analysis for the West Bank

	(1)	(2)	(3)	(4)	(5)
Variables	Food Consumption Score (logged)	Food Insecurity Experience	Economic Hardship	Political Hardship	Agricultural Hardship
Food insecurity experience	−0.0350***				
	(0.00309)				
Economic hardship	−0.0137***	0.434***			
	(0.00517)	(0.0249)			
Agriculture hardship	−0.0195**	0.110***	0.185***		
	(0.00803)	(0.0401)	(0.0247)		
Political hardship	−0.0168	0.0116	0.150***		0.0899***
	(0.0103)	(0.0515)	(0.0318)		(0.0198)
Head of household education (reference: secondary)					
Post-secondary	0.0286**	−0.141**	0.105**	−0.0510**	
	(0.0132)	(0.0660)	(0.0409)	(0.0198)	
Below secondary	0.0182*	−0.0548	−0.0147	−0.00756	
	(0.0108)	(0.0542)	(0.0336)	(0.0163)	
Spouse education (reference: secondary)					
Post-secondary	0.0381***	−0.119*	0.0754*	−0.0424**	
	(0.0128)	(0.0639)	(0.0396)	(0.0192)	
Below secondary	−0.0109	0.0443	−0.00861	−0.0203	
	(0.0104)	(0.0519)	(0.0321)	(0.0156)	
Female head of household	−0.0375**	0.376***	0.0744	−0.0443*	
	(0.0156)	(0.0778)	(0.0481)	(0.0234)	
Registered refugee	−0.0193**	0.0662	0.0457	−0.00203	
	(0.00954)	(0.0477)	(0.0295)	(0.0143)	
Household size	0.0495***	0.430***	0.171***	0.0938***	
	(0.0103)	(0.0509)	(0.0314)	(0.0152)	
Unsustainable	−0.00506	0.533***	0.186***	−0.00741	
	(0.00940)	(0.0463)	(0.0285)	(0.0138)	
Household asset (wealth index)	0.0265***	−0.182***	−0.0541***	−0.0133***	−0.0186***
	(0.00232)	(0.0112)	(0.00692)	(0.00335)	(0.00397)
Aid	5.14e−07	4.40e−06	4.13e−06*	−6.64e−08	
	(7.66e−07)	(3.83e−06)	(2.37e−06)	(1.15e−06)	
Area C	0.0488***	−0.0667	−0.214***	−0.0475*	0.210***
	(0.0168)	(0.0839)	(0.0519)	(0.0251)	(0.0321)
Locality (reference: urban)					
Rural	−0.00602	−0.105**	−0.0597**	0.0570***	0.0433**
	(0.00951)	(0.0475)	(0.0294)	(0.0143)	(0.0184)
Refugee Camp	−0.0591***	0.628***	0.0773	−0.0764***	0.00499
	(0.0177)	(0.0878)	(0.0544)	(0.0264)	(0.0314)
Governorate 1	0.122***	−0.350***	−0.176**	0.0501	0.0335
	(0.0236)	(0.118)	(0.0730)	(0.0355)	(0.0453)
Governorate 2	−0.0299*	0.364***	−0.242***	−0.0477*	−0.145***
	(0.0176)	(0.0877)	(0.0543)	(0.0263)	(0.0338)
Governorate 3	0.0186	0.107	0.149***	−0.0751***	0.0178
	(0.0152)	(0.0759)	(0.0470)	(0.0228)	(0.0292)
Governorate 4	−0.00816	−0.405***	−0.106	−0.0861***	−0.151***
	(0.0209)	(0.104)	(0.0647)	(0.0314)	(0.0402)
Governorate 5	0.0658***	−0.196*	−0.417***	−0.180***	−0.155***
	(0.0234)	(0.117)	(0.0718)	(0.0347)	(0.0446)
Governorate 6	−0.0162	0.274***	−0.232***	−0.119***	0.0600**
	(0.0157)	(0.0783)	(0.0484)	(0.0234)	(0.0300)
Governorate 7	0.000873	−0.324***	0.176**	−0.135***	0.120**
	(0.0245)	(0.122)	(0.0759)	(0.0368)	(0.0474)
Governorate 8	−0.0362*	0.229**	0.118**	0.0291	0.0987***
	(0.0193)	(0.0965)	(0.0598)	(0.0290)	(0.0374)
Governorate 9	−0.0265	0.230***	0.174***	0.200***	0.405***
	(0.0175)	(0.0876)	(0.0542)	(0.0257)	(0.0332)
Governorate 10	−0.0905***	0.401***	−0.0142	−0.0358*	0.159***
	(0.0140)	(0.0696)	(0.0431)	(0.0209)	(0.0266)
Constant	4.313***	0.565***	0.196***	0.0576*	0.144***
	(0.0233)	(0.116)	(0.0720)	(0.0350)	(0.0231)
Observations	4193	4193	4193	4193	4193

Standard errors in parentheses

*p < 0.1, **p < 0.05, ***p < 0.01,

Examining the pathway for food insecurity experience (Model 2) we find that food insecurity is associated with increased economic hardship (*p* < 0.01) and agricultural hardship (*p* < 0.01), female head of household (*p* < 0.01), larger household size (*p* < 0.01), and residence in a refugee camp (*p* < 0.01). Lower level of food insecurity is associated with head of household with post-secondary education (*p* < 0.05), increased household wealth (*p* < 0.01), residence in a rural area (*p* < 0.05).

As economic hardship (Model 3) is associated with both lower dietary diversity and higher level of food insecurity experience, it is critical to examine the impact of economic hardship in the context of food insecurity in a conflict setting. We find that increased agricultural hardship (*p* < 0.01) and political hardship (*p* < 0.01), head of household with post-secondary education (p < 0.05), and larger household size (*p* < 0.01) each have a positive association with economic hardship. Increased household wealth (*p* < 0.01), residence in Area C (*p* < 0.01), and residence in a rural area (*p* < 0.05) are each associated with lower economic hardship. In sum, the economic pathway, which indicates that political hardship and agricultural hardship are positively associated with economic hardship, finds results that are congruent with the literature.

Increased political hardship (Model 4) is associated with larger household size (*p* < 0.01) and residence in a rural area (*p* < 0.05). Head of household (*p* < 0.05) and spouse with post-secondary education (as compared to the reference group of secondary education) (*p* < 0.05), increased household asset aid (*p* < 0.01), and residence in a refugee camp (*p* < 0.01) are associated with a lower level of political hardship.

Increased agricultural hardship (Model 5) in turn is associated with increased political hardship (*p* < 0.01). Similarly, residence in Area C is associated with higher agricultural hardship (*p* < 0.01), which lends support to our hypothesis. However, household wealth has a negative association with agricultural (*p* < 0.01). Of note, aid does not have any statistically significant association with dietary diversity, food insecurity, or any of the hardships examined in this study.

Residence in Area C is found to be associated with higher dietary diversity (*p* < 0.01), which contradicts Hypothesis 2. In these pathways, households who perceived their financial situation to be unsustainable generally reported higher levels of food insecurity experience (*p* < 0.01) and increased economic hardship (*p* < 0.01); this variable does not have a statistically significant association with other outcome variables.

### The Gaza Strip

Most of the relationships between conflict settings, political and economic conditions, and food insecurity from the GS GSEM hold similar patterns to the relationships in the WB GSEM. See Table 4. The dietary diversity pathway in the GS (Model 1) indicates that proximity to a buffer zone in the GS is associated with dietary diversity, indicating that the conflict setting has a direct relationship to reduced dietary diversity. The pathway also shows that food insecurity experience has a negative association with dietary diversity as measured by Food Consumption Score; one unit increase in food insecurity experience is associated with approximately a one-point reduction (natural log of −0.03) in Food Consumption Score (*p* < 0.01). The impact of food insecurity experience on dietary diversity in the GS resembles that in the WB, which provides additional—though not causal nor conclusive—support for our hypothesis that those experiencing food insecurity may lower their dietary diversity to increase caloric intake and cope with food insecurity. The pathway also finds that political refugees (*p* < 0.05), spouse of the head of household with post-secondary education (*p* < 0.01), household size (*p* < 0.01), and household wealth (*p* < 0.01) have positive associations with dietary diversity. On the other hand, proximity to a buffer zone is associated with lower dietary diversity (*p* < 0.01), providing support for Hypothesis 3.1, adding to the literature on how conflict settings reduce dietary diversity.

**Table 4:** GSEM analysis for the Gaza strip

	(1)	(2)	(3)	(4)
VARIABLES	Food Consumption Score (logged)	Food Insecurity Experience	Economic Hardship	Political Hardship
Food insecurity experience	−0.0346***			
	(0.00327)			
Economic hardship	−0.00778*	0.212***		
	(0.00429)	(0.0242)		
Political hardship	0.0109	0.0787**	0.154***	
	(0.00935)	(0.0337)	(0.0258)	
Political refugees	0.0273**			
	(0.0115)			
Registered refugees	0.0286*	0.0933	−0.0212	0.00520
	(0.0158)	(0.0634)	(0.0488)	(0.0353)
Head of household education (reference: secondary)				
Post-secondary	0.0127	−0.159*	0.00495	−0.0364
	(0.0147)	(0.0838)	(0.0645)	(0.0465)
Below secondary	0.00260	0.356***	−0.0329	−0.0338
	(0.0133)	(0.0758)	(0.0584)	(0.0421)
Spouse education (reference: secondary)				
Post-secondary	0.0544***	−0.0598	0.107*	−0.0158
	(0.0144)	(0.0824)	(0.0634)	(0.0458)
Below secondary	−0.0153	0.203***	0.115**	0.128***
	(0.0125)	(0.0712)	(0.0548)	(0.0395)
Female head of household	−0.0274	0.160	−0.0738	0.0671
	(0.0194)	(0.111)	(0.0853)	(0.0616)
Household size	0.0525***	0.724***	0.341***	0.195***
	(0.0127)	(0.0709)	(0.0543)	(0.0390)
Unsustainable	−0.0506***	0.741***	0.320***	0.0183
	(0.0113)	(0.0632)	(0.0483)	(0.0349)
Household asset (wealth index)	0.0309***	−0.271***	−0.0439***	−0.0447***
	(0.00321)	(0.0176)	(0.0135)	(0.00975)
Aid	2.65e−06	−2.58e−05	−3.17e−05*	5.56e−05***
	(3.75e−06)	(2.14e−05)	(1.65e−05)	(1.18e−05)
Proximity to buffer zone	−0.0666***	0.273**	−0.0640	0.683***
	(0.0187)	(0.106)	(0.0817)	(0.0576)
Locality (reference: urban)				
Rural	0.0585***	−0.562***	−0.000941	0.289***
	(0.0210)	(0.119)	(0.0919)	(0.0661)
Refugee camp	−0.00515	0.0821	0.287***	−0.178***
	(0.0144)	(0.0817)	(0.0627)	(0.0451)
Governorate 1	−0.0220	−0.304***	0.506***	−0.122***
	(0.0146)	(0.0830)	(0.0633)	(0.0456)
Governorate 2	0.0276	−0.674***	0.363***	0.179***
	(0.0173)	(0.0980)	(0.0751)	(0.0542)
Governorate 3	−0.0839***	−0.411***	−0.0480	−0.101**
	(0.0157)	(0.0891)	(0.0686)	(0.0495)
Governorate 4	−0.0476***	−0.180*	−0.393***	0.162***
	(0.0176)	(0.100)	(0.0770)	(0.0555)
Constant	4.290***	0.780***	0.342***	0.424***
	(0.0300)	(0.165)	(0.127)	(0.0915)
Observations	2888	2888	2888	2888

Standard errors in parentheses

*p < 0.1, **p < 0.05, ***p < 0.01

The pathway for food insecurity experience (Model 2) shows that proximity to a buffer zone has statistically significant associations with increased food insecurity experience, supporting Hypothesis 3.1 that conflict settings increase food insecurity. Increased food insecurity experience also is associated with increased economic hardship (*p* < 0.01) and political hardship (*p* < 0.05) and head of household (*p* < 0.01) and spouse (*p* < 0.01) with below secondary education, and household size (*p* < 0.01). On the other hand, household wealth (*p* < 0.01) and locality type (*p* < 0.01) each has a negative association with economic hardship.

Model 3 includes the pathway for economic hardship. The results indicate that increased economic hardship is directly associated with increased political hardship (*p* < 0.01), spouse of head of household with below secondary education (*p* < 0.05), and larger household size (*p* < 0.01). As expected, increased household wealth is associated with reduced economic hardship (*p* < 0.01).

Model 4 presents the findings for the pathway for political hardship. We find that proximity to a buffer zone is associated with political hardship, with those residing within one kilometer of buffer zone experiencing near one additional item of political hardship compared to those living more than one kilometer away. Spouse of head of household with below secondary education (as compared to having secondary education) (*p* < 0.01) and larger household size (*p* < 0.01) are associated with increased political hardship. Aid is also associated with political hardship, but the substantive effect is incredibly small at 0.00006 (*p* < 0.01). Increase household wealth is the only variable to be associated with reduced political hardship (*p* < 0.01). In summary, the GSEM model for the GS suggests that living in close proximity to a buffer zone is associated with higher level of economic and political hardships and these hardships are in turn associated with food insecurity experience and lower food diversity.

## Discussion

This study demonstrates that previously identified political, economic, and agricultural determinants for food insecurity have an interactive impact on food insecurity. Furthermore, we highlight the association between different measures of food insecurity and add to the evidence that individuals in conflict-affected settings may adopt coping strategies to maximize their caloric intake.

Political, economic, and agricultural hardships have a significant association and potential impact on aspects of food insecurity in the oPt, which has been experiencing a prolonged Israeli military occupation. In the WB, residents of Area C experience a higher level of agricultural hardship. While residing in Area C is not directly associated with food insecurity experience, the findings suggest that the political conditions in Area C may indirectly increase food insecurity experience through exerting agricultural hardships. Concomitantly, agricultural hardship is directly associated with lower dietary diversity and a higher level of food insecurity experience. In other words, living in Area C may not be associated directly with insecurity experience, but the hardships generated by conditions in Area C may increase food insecurity experience.

The analyses also suggest that residing in Area C is associated with higher dietary diversity, even when controlling for hardships. We reason that this statistical finding may be due to the fact those residing in Area C but are not working in the agricultural sector are likely to travel outside of Area C for employment. As such, they may be less impacted than those working in the agricultural sector.

In the analysis for the GS, the potential impact of the conflict on food insecurity is evident and severe throughout the hypothesized pathways. We find that living in close proximity to a buffer zone not only is associated directly with lower dietary diversity and a higher level of food insecurity experience, but also is associated with political hardship—which in turn is associated with increased food insecurity experience. This finding further underlines the harmful living condition in the area; buffer zones are closely monitored and patrolled by Israeli soldiers, and Palestinians who need to access land close to a buffer zone often suffer harassment and report difficulties. Furthermore, the experience of food insecurity is associated with a one-point reduction in dietary diversity, indicating that those experiencing food insecurity in the GS also have lower quality diets or may be engaging in a coping mechanism to maximize caloric intake in a condition with insufficient food.

Our analyses show that living conditions associated with the protracted conflict and military occupation in the oPt directly increased hardships and the experience of food insecurity and are detrimental to dietary diversity. More broadly, our study demonstrates the inverse relationship between food insecurity experience and dietary diversity in a conflict-affected setting. Increased food insecurity experience is associated with lower dietary diversity in conflict-affected settings likely because food insecurity households may choose to consume more high caloric food that are affordable.

These results are especially policy relevant as nearly a third of the population in the oPt is estimated to be experiencing food insecurity. The prevalence of moderate or severe food insecurity in the population was 29.9% between 2014 and 2016—with 9.5% experiencing severe food insecurity [29]. As a result, children face stunting—with height-for-age at 7.4% for children under the age of five  —and wasting—with weight-for height at 1.2% [34]. The findings here should be carefully considered when formulating foreign and humanitarian policies for Palestinians living the WB and the GS. Decision-makers should be cognizant of the role political hardship, economic hardship, and agricultural hardship play in food insecurity as well as the manner to which the population has been severely impacted by the protracted conflict and occupation.

Our study indicates that aid has minimal potential impact on reducing food insecurity and mitigating hardships—despite the fact that international communities have devoted resources and effort into humanitarian aid [71]. This finding suggests that it may be beneficial for aid programs to provide additional resource, such as agricultural assistance. Agricultural assistance in prolong conflicts may stimulate the economy, decrease the level of unemployment and dependency in aid, and reduce food insecurity. Additionally, it may be beneficial for aid programs to provide resources to support infrastructural development in conflict-affected settings. Infrastructural developments such as water treatment facilities can work in conjunction with agricultural assistance to sustain the agricultural industry during conflict and increase food production.

The highlighted pattern also suggests that it may be necessary for international communities to consider the negative impact of political hardship on food insecurity as well as on the effectiveness of aid programs established to mitigate food insecurity. In this study, political hardship is operationalized by loss in assets or projects due to Israeli measures, restriction imposed on access to land, and lack of permits. The strong associations between political hardship, other hardships, and food insecurity experience and lower dietary diversity, indicate that political hardship is likely so severe in the context of the oPt that it not only worsens food insecurity but also impedes the valuable benefits of aid programs.

Political hardship is highly relevant when considering the importance of access to aid and coverage for all as well as human justice. Aid access, similar to healthcare access, does not imply coverage of all cases for all people. Access is attained when people are provided with the opportunity and ability to obtain the services and coverage for all is when people can actually obtain the services they need. In the current context, it has been difficult to ensure both access and coverage for all in the oPt. For example, due to home destructions, around 100,000 were internally displaced persons (IDP) in the oPt and only one third of IDP receive housing support. And, while IDP should receive a temporary shelter cash assistance (TSCA) of US$200–250 per month per family, only 41% of surveyed families reported receiving TSCA on regular basis, 13% were uncertain about their current eligibility, and the rest has had their payment stopped or had never received any payment [59].

Aid programs should combine immediate survival needs with calls for justice to Palestinians. Previous literature has pointed out that aid in the oPt often stops at contingent and ad hoc interventions, including only material provision. The interaction between political, economic, and agriculture factors confirms that broader structural factors play an important role in affecting food insecurity. Consequently, interventions to ameliorate food insecurity need to take place on a much higher level than only food assistance. As such, it is critical for the actors in the international community to call for the removal of political obstacles detrimental to the lives of those residing in the oPt.

## Conclusions

Households and persons residing in conflict-affected areas are often found to rely on less preferred foods and to limit the variety of foods eaten and the portion size of meals consumed. While previous studies examine the impact of conflict on different food security measures, the relationship between these measures as well as their relationship with political, economic, and agricultural factors remain under explored. This study examines the association between political, economic, and agricultural factors, and food insecurity experience and dietary diversity in a conflict-affected setting—that of the occupied Palestinian territory. We find in the West Bank, residency in Area C is associated with a higher level of agricultural hardship and a higher dietary diversity. In the Gaza Strip, living within one kilometer to a buffer zone is associated with lower dietary diversity, higher level of political hardship, and higher levels food insecurity experience.

The conflict and living conditions in the occupied Palestinian territory provide an opportunity to evaluate pathways to food insecurity, political, economic, and agricultural hardships, and potential coping mechanismsl; however, this is only a start. More comprehensive surveys need to be conducted and data need to be collected to examine the relationship and nuances between the interaction of political, economic, and agricultural factors and aspects of food security in conflict-affected settings. Furthermore, the question whether individuals were engaging the coping mechanisms need to be further explored with studies focusing on understanding the potential trade-offs that are involved. We hope future studies build on the findings in this study and evaluate the causal arrow in the context of conflict, political, economic, and agricultural hardships, and aspects of food security.

Understanding the intricacies in relationships described and analyzed above is especially critical for policy making; while food insecurity may be an externality of conflict it may also be a direct strategy to discourage residency in contested territories or territories under threat of annexation. Political determinants are critically intertwined and associated with different aspects of food security. Therefore, it is crucial to ameliorate the current political and economic structures, introduce sustainable interventions to minimize food insecurity, and provide humanitarian intervention and aid support with full coverage.

## Data Availability

The datasets used and/or analysed during the current study are available from the corresponding author on reasonable request.
